# CT Texture Patterns Reflect HPV Status but Not Histological Differentiation in Oropharyngeal Squamous Cell Carcinoma

**DOI:** 10.3390/cancers17142317

**Published:** 2025-07-11

**Authors:** Lays Assolini Pinheiro de Oliveira, Caio Elias Irajaya Lobo Peresi, Daniel Vitor Aguiar Nozaki, Ericka Francislaine Dias Costa, Lana Ferreira Santos, Carmen Silvia Passos Lima, Sérgio Lúcio Pereira de Castro Lopes, Andre Luiz Ferreira Costa

**Affiliations:** 1 Department of Anesthesiology, Oncology and Radiology, Faculty of Medical Sciences, University of Campinas (UNICAMP), Campinas 13083-887, SP, Brazil; lays_08@hotmail.com (L.A.P.d.O.); caioperesi@hotmail.com (C.E.I.L.P.); carmenl@unicamp.br (C.S.P.L.); 2 Postgraduate Program in Dentistry, Dentomaxillofacial Radiology and Imaging Laboratory, Department of Dentistry, Cruzeiro do Sul University (UNICSUL), São Paulo 01506-000, SP, Brazil; 3 School of Dentistry of Paulista Association of Dentists (FAOA), São Paulo 01310-000, SP, Brazil; daniel.odontofaoa@gmail.com; 4 Laboratory of Cancer Genetics, School of Medical Sciences, University of Campinas (UNICAMP), São Paulo 13083-872, SP, Brazil; erickaf@unicamp.br; 5 Department of Diagnosis and Surgery, The Institute of Sciences and Technology of São Paulo State University (UNESP), São José dos Campos 12245-000, SP, Brazil; lana.santos@unesp.br (L.F.S.); sergioluciolopes@gmail.com (S.L.P.d.C.L.)

**Keywords:** oropharyngeal squamous cell carcinoma, texture analysis, radiomics, imaging biomarkers, non-invasive diagnosis

## Abstract

Background: Texture analysis (TA) has shown promise in characterizing intratumoral heterogeneity from imaging data. We add to the literature that shows its capability to differentiate oropharyngeal cancers based on HPV status. Methods: Multislice CT analysis was done in 120 patients with confirmed OP SCC: a single 5 mm region of interest was placed on three consecutive homogeneous CT slices per patient. Texture features were extracted by using gray-level co-occurrence matrices averaged per patient. HPV status (via p16 IHC and molecular confirmation) and differentiation grade (i.e., good, moderate, and poor) were recorded. Non-parametric statistical tests assessed differences between subgroups. Results: Seven texture parameters (i.e., angular second moment, contrast, sum of squares, sum entropy, entropy, inverse difference moment, and difference variance) differed significantly between HPV+ and HPV− tumors (all *p* < 0.05). HPV+ tumors exhibited increased heterogeneity and complexity on CT imaging. No texture feature correlated with histological grade. Conclusions: This study adds to the growing evidence that CT-based TA can assess HPV status in OP SCC. TA may be promising, though it requires further validation as an adjunctive method integrating into radiomics workflows to develop predictive models for diagnosis, prognosis, and treatment planning.

## Simple Summary

We applied texture analysis (TA) to CT scans of oropharynx (OP) squamous cell carcinoma (SCC) to explore whether imaging-derived features could differentiate tumors based on HPV status and histological grade. We found seven specific TA parameters that could distinguish HPV+ from HPV− tumors, but none correlated with differentiation grade.

## 1. Introduction

Oropharyngeal squamous cell carcinoma (OPSCC) is an increasingly common malignancy largely driven by infection with human papillomavirus (HPV), particularly HPV-16 [1,2]. HPV+ OPSCC compared with HPV− impacts a younger population, has a better prognosis, and is more sensitive to irradiation [3]. Guidelines by the American Society of Clinical Oncology (ASCO) and the College of American Pathologists recommend HPV detection through nucleic acid-based molecular techniques, such as polymerase chain reaction (PCR) and in situ hybridization (ISH), often in combination with immunohistochemical (IHC) expression of p16 as a surrogate marker [4].

Radiomics is a rapidly growing field in cancer imaging that involves the extraction of high-dimensional quantitative features from medical images that characterize tumor morphology, texture, and heterogeneity in a non-invasive manner [5]. Among its applications, texture analysis (TA) derived from computed tomography (CT) (and MRI and PET-CT) has shown promise in differentiating HPV+ and HPV− OPSCC by quantifying intratumoral complexity and spatial distribution of grayscale intensities [6,7,8,9]. Imaging assesses tumor extent and is combined with clinical evaluation and histopathology [10,11,12,13]. Prior studies have investigated radiomic signatures associated with HPV status [6,7], often focusing on classification performance or machine learning pipelines.

However, most previous studies have focused on distinguishing HPV status or predicting outcomes using radiomics, often through advanced classification models, without concurrently assessing traditional histopathological factors such as tumor differentiation. Evaluating texture features in relation to both HPV status and histological grade provides an opportunity to expand the understanding of how these imaging parameters may contribute to characterizing OPSCC.

The aim of this study was to add to the existing literature investigating texture features from pre-treatment CT scans in OPSCC patients defined by HPV status and histological tumor differentiation.

## 2. Materials and Methods

### 2.1. Ethical Approval

This retrospective observational study was conducted in accordance with the ethical standards of the institutional research committee and the 1964 Helsinki Declaration and its later amendments. The study was approved by the Research Ethics Committee of the Faculty of Medical Sciences of the University of Campinas (FCM-UNICAMP), Campinas, São Paulo, Brazil, under protocol number CAE: 33377320.0.0000.5404.

### 2.2. Patient Selection

One hundred and thirty-two patients with histopathologically confirmed OPSCC were screened through the database of the Clinics Hospital of UNICAMP. After applying eligibility criteria, 120 patients (age 38–85 years; 88% male) were included. All had undergone preoperative contrast-enhanced multislice CT, and clinical records were reviewed for demographic and clinical data. The inclusion criteria were availability of preoperative contrast-enhanced multislice CT, histopathological confirmation of OPSCC with classification into well, moderately, or poorly differentiated grades, and determination of HPV status. The exclusion criteria included images with significant artifacts and incomplete or inconclusive medical records. Informed consent was obtained from all living patients; the requirement for consent was waived in accordance with local regulations for deceased patients.

### 2.3. HPV Detection

HPV status was determined using p16 immunohistochemistry (IHC) and nucleic acid-based techniques, as described by Singhi and Westra [14,15]. Formalin-fixed, paraffin-embedded tumor samples were first tested by using p16 IHC, and those with strong nuclear and cytoplasmic staining in ≥70% of the tumor cells were considered p16+. These samples were then confirmed using polymerase chain reaction (PCR), in situ hybridization (ISH), or RNA-ISH targeting high-risk HPV genotypes. Only cases positive in both p16 IHC and molecular testing were classified as HPV+.

### 2.4. Lymph Node Staging

Nodal staging was per the TNM classification system (8th Ed AJCC/UICC) [16]. Stages I, III, Iva, and IVb were defined based on primary tumor extension and extent and laterality of lymph node metastases, with N-stage based on radiological interpretation by two experienced radiologists.

### 2.5. CT Image Acquisition and Processing

All patients underwent contrast-enhanced multislice CT on a 64-slice scanner (Aquilion, Toshiba Medical Systems Corporation, Otawara, Japan). Scans were performed according to the following parameters: 120 kVp, 400 mA, 512 × 512 matrix, 3 mm slice thickness, 3 mm reconstruction interval, and 320-mm field of view (FOV). Images were acquired in the axial plane and stored in DICOM (Digital Imaging and Communications in Medicine) format.

**Figure 1:**
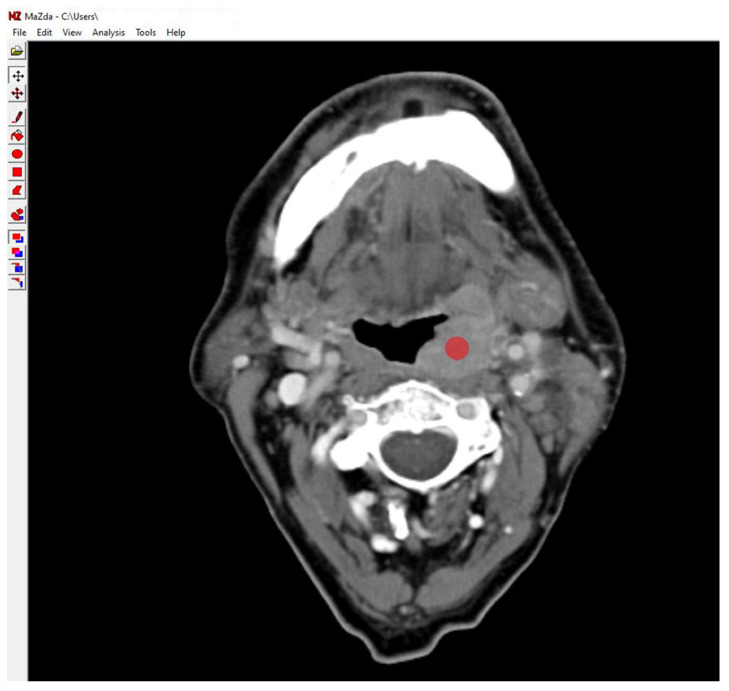
Example of contrast-enhanced axial multislice CT image displayed in the MaZda software interface, showing manual placement of a 5 mm circular region of interest (ROI) within a homogeneous area of the tumor.

Image visualization and preprocessing were performed on a Windows^®^-based system (Dell^®^, Round Rock, TX, USA, Intel Core i7 processor, 64-bit, Intel Corporation, Santa Clara, CA, USA). For standardization, three representative axial slices from each tumor were selected based on maximum lesion extent and clarity [17,18]. A single circular region of interest (ROI) with a diameter of 5 mm was manually positioned in a homogeneous area of the tumor on the axial contrast-enhanced CT slices by using the OnDemand3D™ software, version 1.0, (CyberMed Inc., Seoul, Republic of Korea). The ROI was carefully placed to avoid areas of necrosis, calcification, or image artifacts, thus ensuring the inclusion of a representative portion of the viable tumor tissue. Two experienced observers, both trained in head and neck radiology, jointly selected the ROI for each case by consensus to ensure reproducibility. The selected images were then exported in bitmap (BMP) format for texture feature extraction. An example of ROI placement in the MaZda software (version 4.6) interface is shown in Figure 1.

### 2.6. Texture Feature Extraction

TA was conducted by using the MaZda software package (http://www.eletel.eu/mazda) accessed on 2 April 2025, which enables extraction of Haralick texture features from gray-level co-occurrence matrices (GLCM). The texture parameters of each of the three slices were extracted, and the average value was calculated and considered as an individual value for each patient [17,18]. Eleven second-order texture features were extracted as follows: angular second moment (AngScMom), contrast (Contrast), correlation (Correlat), sum of squares (SumOfSqs), inverse difference moment (InvDfMom), entropy (Entropy), sum average (SumAverg), sum variance (SumVarnc), sum entropy (SumEntrp), difference variance (DifVarnc), and difference entropy (DifEntrp) [19,20].

Images were normalized to 128 gray levels prior to feature extraction. Each parameter was calculated in four directions (i.e., 0°, 45°, 90°, and 135°) and at pixel distances from 1 to 3 [17,18,19]. The final value for each texture feature was obtained by averaging the measurements across the three slices and all directions, thus enhancing the volumetric representativeness and robustness of the texture data [17,18].

### 2.7. Statistical Analysis

Since the texture features did not follow a normal distribution, non-parametric tests were applied. Comparisons between more than two groups were performed by using the Kruskal–Wallis test, while pairwise comparisons were conducted by using the Mann–Whitney U test. A significance level of 5% was adopted for all analyses. Statistical computations were performed by using R software, version 3.6.0 (The R Foundation for Statistical Computing, Vienna, Austria); (https://www.r-project.org).

## 3. Results

One hundred twenty subjects of both genders (88% males), aged between 38 and 85 years old (mean age of 57 years), participated in the study. The patients were classified according to HPV status (i.e., positive or negative) and cell differentiation grade (i.e., poor, moderate, and good). All primary tumors were located within the oropharynx, involving regions such as the tonsillar pillars and fossa, base of tongue, soft palate, and lateral oropharyngeal walls. The distribution of T stages did not differ significantly between HPV-positive and HPV-negative groups (*p* > 0.05), indicating comparable primary tumor sizes across the cohorts.

**Figure 2:**
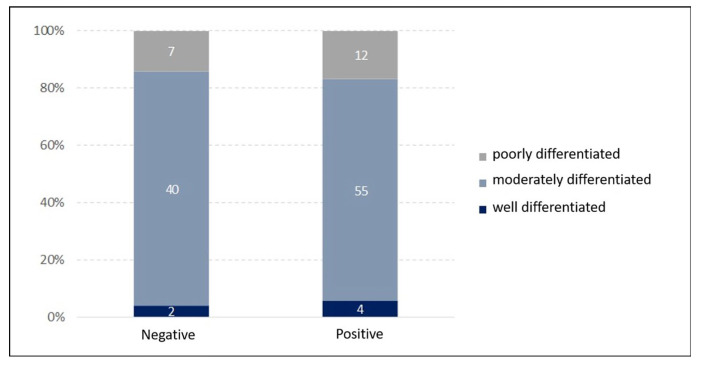
Relationship between HPV status and differentiation grade (*p*-value = 0.849).

Below, Figure 2 shows the association between these two types of classification. One can note that the percentage distribution of differentiation grades is similar between both groups of HPV status. No statistically significant association was found between HPV status and differentiation grade (*p*-value = 0.849; chi-square test).

Below, Table 1 shows the profile of the patients (i.e., smoking, alcohol use, gender and clinical stage) per HIV group and cell differentiation grade.

**Figure 3:**
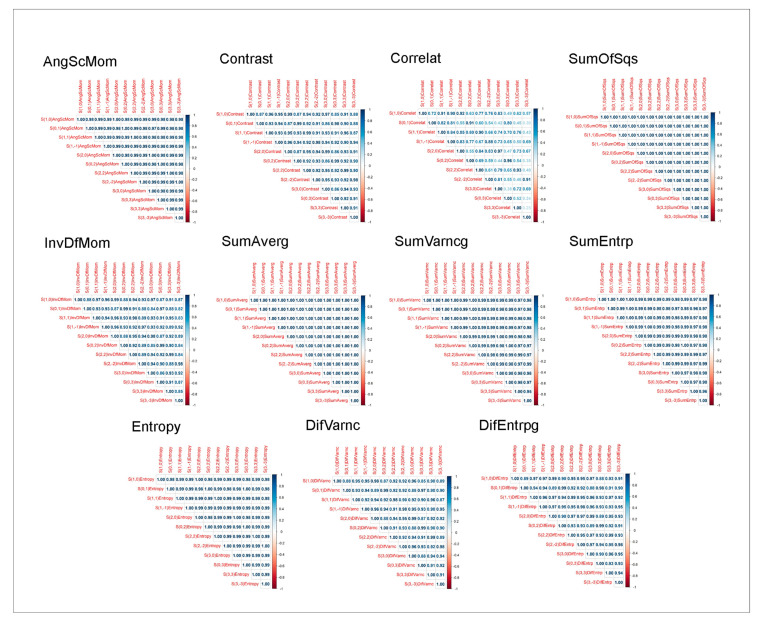
Heatmaps of Spearman’s correlation coefficients between distances for each texture parameter derived from gray-level co-occurrence matrices (GLCM). The analysis was performed separately for angular second moment (AngScMom), contrast, correlation (Correlat), sum of squares (SumOfSqs), inverse difference moment (InvDfMom), sum average (SumAverg), sum variance (SumVarNc), sum entropy (SumEntrp), entropy, difference variance (DifVarNc), and difference entropy (DifEntrp). Each heatmap illustrates the consistency of texture values across pixel distances within the analyzed CT images.

TA yielded 11 parameters calculated across five different directions. Statistical analysis of such a large number of variables significantly increases the likelihood of type I errors. To mitigate this, the number of variables was reduced by calculating the mean across the five directions. Prior to averaging, Spearman’s correlation coefficients were computed between directions. High correlation was observed for 10 out of the 11 parameters, justifying the use of the mean values. However, the correlation between directions was not high for the parameter Correlat, which required individual assessment across directions (Figure 3).

**Figure 4:**
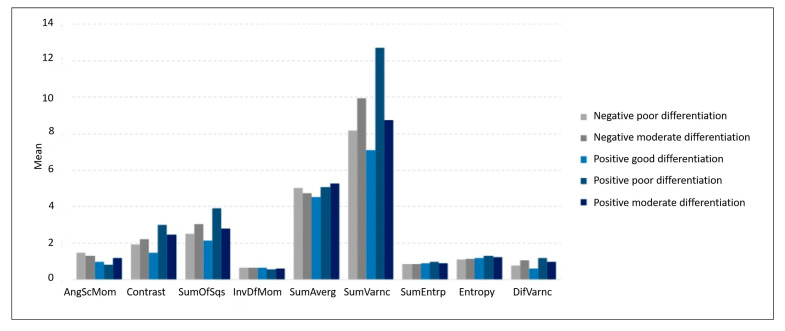
Mean values of texture parameters according to HPV status and histological differentiation groups. To allow graphical comparison on a common scale, the mean value of angular second moment (AngScMom) was multiplied by 10, and the mean value of sum average (SumAverg) was divided by 10.

**Figure 5:**
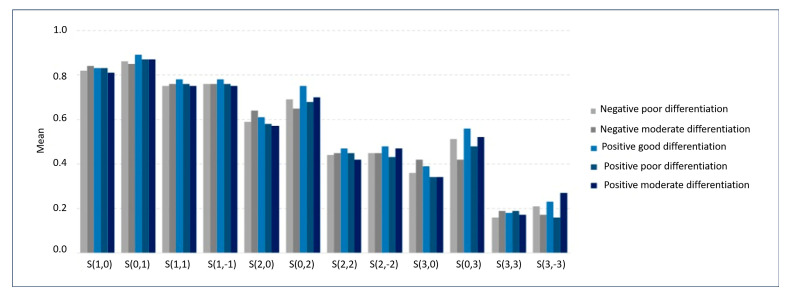
Mean values of the parameter Correlation (Correlat), calculated for each directional pair (distance and angle) across HPV status and histological differentiation groups.

In the comparison between the groups, good differentiation for HPV-negative status was not considered because this group had two subjects only. Table 2 and Table 3, as well as Figure 4 and Figure 5, show the comparison between the groups for texture parameters.

One can observe that the HPV-negative group with moderate differentiation showed a higher value of AngScMom compared to that of the HPV-positive group with moderate differentiation (*p*-value = 0.038).

By comparing the groups of cell differentiation only, without separation per HPV status, it was observed that there was no statistically significant difference between the groups (Table 4).

Table 5 shows the comparison between the groups of HPV status without separation per differentiation grade.

Table 5 shows that statistically significant differences were found between HPV-positive and HPV-negative groups regarding the following texture parameters: AngScMom; Contrast; SumOfSqs; SumEntrp; Entropy and DifVarnc (with the HPV-positive group having higher values compared to the HPV-negative group) and InvDfMom (with the HPV-positive group having lower values compared to the HPV-negative group). Parameter SumVarnc (*p* = 0.044) showed a statistical trend towards differentiation as the HPV-positive group had higher values.

## 4. Discussion

Advances in image post-processing methodologies such as texture analysis (TA) enable quantification of complex image structures [17,18,19,20]. TA is a mathematical method used for assessment and relationships/distribution of adjacent gray levels, which can assist in tissue characterization [21,22]. It has been applied in evaluating head and neck tumors, characterizing tumor heterogeneity, and correlating with biological behavior. Studies using CT-based TA in head and neck SCC have demonstrated potential to differentiate histopathological subtypes and assess prognosis of various malignancies [23,24,25]. This supports growing interest in using non-invasive image-derived biomarkers to improve diagnostic accuracy and guide treatment strategies in oncologic imaging.

Multislice CT images in this study of OPSCC found no statistically significant differences with respect to differentiation among the 11 texture parameters analyzed [17], but did find correlation with HPV status in 7 of 11: angular second moment (Ang ScMom), contrast, sum of squares (SumOfSqs), sum entropy (SumEntrp), entropy, inverse difference moment (InvDfMom), and difference variance (DifVarnc). These reflect heterogeneity, uniformity, and complexity in the CT images.

AngScMom showed significantly lower values in HPV+ cases (*p* = 0.003), indicating greater image heterogeneity. This parameter reflects image uniformity or energy, whereas lower values suggest increased disorder. A similar result was reported in a study in which TA was used to differentiate periapical cysts and granulomas in CT images [19], where AngScMom was also significantly lower in granulomas due to their heterogeneous granulation tissue, resembling the behavior observed in HPV-positive tumors in our study. Contrast was also significantly higher in HPV+ tumors (*p* = 0.016), indicating increased local pixel intensity variation and less homogeneity. These findings align with those by Costa [18], who observed high contrast values in non-odontogenic sinusitis due to heterogeneous sinus content, reflecting the irregularity observed in HPV+ tumors.

SumOfSqs, associated with gray-level dispersion, was significantly higher in the HPV+ group (*p* = 0.034), suggesting a greater variability potentially due to necrosis or cellular heterogeneity. Entropy and SumEntrp were higher in HPV+ tumors (*p* = 0.0005 and *p* = 0.008), reflecting greater structural complexity and internal disorder. These results are consistent with studies on osteonecrosis [26] and implant stability [18], where increased entropy indicated tissue disorganization.

InvDfMom and DifVarnc also showed significant differences (*p* = 0.006 and *p* = 0.024, respectively). InvDfMom was lower in HPV-positive tumors, indicating reduced homogeneity, whereas DifVarnc was higher, suggesting increased internal irregularities. A prior study [27] using TA to differentiate pleomorphic adenoma from Warthin tumor also found DifVarnc, InvDfMom, entropy, SumEntrp, and contrast indicated internal disorganization, similar to our findings in HPV+ tumors.

Taken together, the behavior of these texture parameters demonstrates that HPV+ tumors impact histological and imaging characteristics, exhibiting patterns of greater heterogeneity, disorganized cellular proliferation with necrosis and with greater angiogenesis [28], and internal variability in multislice CT images, as indicated by the higher values of contrast, entropy, and DifVarnc. A study by Mungai et al. [8] evaluated the association between tumor heterogeneity and HPV status in OPSCC using contrast-enhanced CT images. They identified differences primarily in higher-order texture features derived from GLRLM (Gray-Level Run-Length Matrix), GLZLM (Gray-Level Zone Length Matrix), and NGLDM (Neighborhood Gray-Level Different Matrix) matrices, although no significant differences were found in GLCM-based parameters. In contrast, the present study demonstrated significant differences in seven GLCM-derived parameters. These discrepancies may result from differences in segmentation strategy (whole-tumor vs. standardized ROI in homogeneous regions) or feature extraction methods (a large set of higher-order features in Mungai et al. vs. a focused set of GLCM-derived parameters). Both studies, however, support the role of CT-based TA as a promising approach to characterize HPV-related tumor heterogeneity in OPSCC. Our findings are partially consistent with those reported by Lee et al. [25], who demonstrated that CT TA could distinguish HPV status in OPSCC by capturing differences in tumor heterogeneity. In their study, HPV+ tumors exhibited lower energy and higher entropy values, reflecting greater textural complexity and less uniformity, which are also parameters significantly different compared to our results. Although both studies highlight the diagnostic potential of texture features (e.g., entropy and contrast) in assessing HPV-related changes, our approach differed in that a smaller, standardized ROI was used in homogeneous tumor regions and values were averaged across multiple slices, which may have influenced the specific parameters found to be significant. Nevertheless, the convergence in the identification of higher heterogeneity in HPV+ tumors reinforces the notion that radiomic texture biomarkers reflect underlying biological behavior.

In addition to CT-based investigations, prior studies have explored the role of texture analysis in OPSCC and head and neck SCC using other imaging modalities such as MRI and PET-CT. For example, Liao et al. [29] demonstrated that radiomic features extracted from PET images could differentiate metabolic patterns in oropharyngeal and hypopharyngeal cancers, highlighting the potential of PET-based TA for characterizing tumor biology beyond anatomical imaging. Similarly, Meyer et al. [30] correlated CT-derived texture parameters with histopathological features in head and neck SCC, emphasizing the relationship between imaging heterogeneity and underlying tumor architecture. Kim et al. [31] used CT TA to discriminate tonsil cancers from normal palatine tonsils, supporting the application of TA even in early detection scenarios. Furthermore, a comprehensive systematic review by Bicci et al. [9] summarized multiple studies employing TA in OPSCC, including investigations using CT, MRI, and PET, underscoring its versatility across modalities for assessing HPV status, tumor grade, recurrence risk, and treatment response. These prior works reinforce the concept of texture analysis as a valuable non-invasive imaging tool for evaluating tumor heterogeneity, which complements the findings of our study focused on contrast-enhanced multislice CT in OPSCC.

Importantly, although we demonstrated statistically significant differences in texture parameters between HPV-positive and HPV-negative tumors, we did not calculate predictive metrics such as sensitivity, specificity, positive predictive value (PPV), or negative predictive value (NPV). These measures are necessary to determine the actual clinical utility of TA as a diagnostic adjunct. Future studies will involve developing predictive models (e.g., using ROC curves) to establish cutoffs and compute these metrics, thus providing a stronger foundation for clinical translation.

Despite these promising results, several limitations should be acknowledged. First, the retrospective design and single-institution dataset may restrict the generalizability of the findings. Second, although all tumors were located within the oropharynx, the study did not stratify cases by specific subsites such as tonsil, base of tongue, or soft palate, which could influence HPV prevalence and texture characteristics. Third, while T stage distribution was similar between groups, subtle differences in primary tumor size may still have impacted texture measurements. Additionally, the relatively small, standardized ROI approach (though reproducible) may not capture the full extent of tumor heterogeneity compared to whole-tumor segmentation. Only first-order and GLCM-derived texture features were analyzed, and more advanced radiomic descriptors might offer further insights. Finally, the absence of an external validation cohort warrants caution when extrapolating these results to broader clinical settings.

Future prospective studies with larger, multicenter datasets are needed to validate the clinical utility of CT-based texture biomarkers in OPSCC. In addition, incorporating machine learning and deep learning techniques to automate segmentation and classification could minimize operator dependence and enhance clinical workflow efficiency. Exploring whether these texture-derived biomarkers maintain predictive performance across other head and neck subsites (where the prognostic significance of HPV differs) may further broaden their applicability in oncologic imaging.

## 5. Conclusions

This study adds support to the literature that multislice CT TA quantitatively differentiates OPSCC based on HPV status: HPV+ tumors exhibit greater heterogeneity and complexity. This approach, though requiring further validation for routine clinical implementation, may contribute to improving non-invasive characterization of tumor behavior and may guide future developments in predictive models for diagnosis, prognosis, and treatment planning.

## Figures and Tables

**Table 1:** Profile of the patients per HPV groups and cell differentiation grade.

Variable	Negative HPV Status			Positive HPV Status		
	Good Differentiation (n = 2)	Moderated Differentiation (n = 40)	Poor Differentiation (n = 7)	Good Differentiation (n = 4)	Moderated Differentiation (n = 55)	Poor Differentiation (n = 12)
Smoking:						
No	0	2	1	0	6	3
Yes	2	38	6	4	49	9
Alcohol use:						
No	0	6	0	1	11	2
Yes	2	34	7	3	44	10
Gender:						
Female	0	8	0	0	5	1
Male	2	32	7	4	50	11
Clinical stage:						
I	1	1	0	0	1	1
II	0	0	0	0	2	0
III	0	7	0	1	9	2
IVA	1	20	5	1	24	6
IVB	0	8	1	1	12	1
IVC	0	4	1	1	6	2
X	0	0	0	0	1	0

**Table 2:** Mean and standard deviation of the TA parameters per HPV status group and cell differentiation.

Variable	Negative		Positive		
	Moderate Differentiation (n = 40)	Poor Differentiation (n = 7)	Good Differentiation (n = 4)	Moderate Differentiation (n = 55)	Poor Differentiation (n = 12)
AngScMom	0.15 (0.14)	0.13 (0.07)	0.10 (0.05)	0.08 (0.07)	0.12 (0.11)
Contrast	1.93 (2.12)	2.22 (2.15)	1.47 (0.73)	3.00 (3.18)	2.49 (2.88)
S (1, 0) Correlat	0.82 (0.08)	0.84 (0.03)	0.83 (0.12)	0.83 (0.10)	0.81 (0.10)
S (0, 1) Correlat	0.86 (0.06)	0.85 (0.05)	0.89 (0.03)	0.87 (0.08)	0.87 (0.06)
S (1, 1) Correlat	0.75 (0.10)	0.76 (0.05)	0.78 (0.13)	0.76 (0.12)	0.75 (0.12)
S (1, −1) Correlat	0.76 (0.10)	0.76 (0.06)	0.78 (0.12)	0.76 (0.13)	0.75 (0.15)
S (2, 0) Correlat	0.59 (0.18)	0.64 (0.09)	0.61 (0.29)	0.58 (0.21)	0.57 (0.23)
S (0, 2) Correlat	0.69 (0.12)	0.65 (0.07)	0.75 (0.07)	0.68 (0.14)	0.70 (0.14)
S (2, 2) Correlat	0.44 (0.19)	0.45 (0.10)	0.47 (0.30)	0.45 (0.22)	0.42 (0.20)
S (2, −2) Correlat	0.45 (0.20)	0.45 (0.09)	0.48 (0.24)	0.43 (0.19)	0.47 (0.25)
S (3, 0) Correlat	0.36 (0.24)	0.42 (0.12)	0.39 (0.36)	0.34 (0.25)	0.34 (0.26)
S (0, 3) Correlat	0.51 (0.15)	0.42 (0.11)	0.56 (0.12)	0.48 (0.18)	0.52 (0.19)
S (3, 3) Correlat	0.16 (0.25)	0.19 (0.11)	0.18 (0.33)	0.19 (0.25)	0.17 (0.19)
S (3, −3) Correlat	0.21 (0.23)	0.17 (0.08)	0.23 (0.18)	0.16 (0.19)	0.27 (0.27)
SumOfSqs	2.52 (2.94)	3.04 (3.41)	2.14 (1.56)	3.91 (5.43)	2.81 (2.17)
InvDfMom	0.67 (0.14)	0.67 (0.12)	0.65 (0.09)	0.59 (0.13)	0.63 (0.15)
SumAverg	50.3 (12.7)	47.5 (11.6)	45.3 (12.0)	50.8 (10.8)	52.8 (11.8)
SumVarnc	8.16 (10.1)	9.93 (11.5)	7.10 (5.71)	12.7 (18.8)	8.76 (6.31)
SumEntrp	0.85 (0.24)	0.87 (0.19)	0.92 (0.14)	0.97 (0.19)	0.92 (0.21)
Entropy	1.12 (0.38)	1.14 (0.30)	1.20 (0.21)	1.33 (0.32)	1.24 (0.36)
DifVarnc	0.78 (0.73)	1.05 (1.07)	0.62 (0.27)	1.18 (1.17)	0.99 (1.02)

Note: angular second moment (AngScMom), contrast (Contrast), correlation (Correlat), sum of squares (SumOfSqs), inverse difference moment (InvDfMom), entropy (Entropy), sum average (SumAverg), sum variance (SumVarnc), sum entropy (SumEntrp), difference variance (DifVarnc), and difference entropy (DifEntrp).

**Table 3:** Median, first and third quartiles of the TA parameters and *p*-value for comparison between groups (Kruskal–Wallis test).

Variable	Negative		Positive			*p*-Value
	Moderate Differentiation (n = 40)	Poor Differentiation (n = 7)	Good Differentiation (n = 4)	Moderate Differentiation (n = 55)	Poor Differentiation (n = 12)	
AngScMom	0.09 [0.06; 0.18]	0.13 [0.08; 0.17]	0.09 [0.08; 0.11]	0.07 [0.04; 0.12]	0.08 [0.06; 0.15]	0.038
Contrast	1.26 [0.63; 2.31]	0.96 [0.73; 3.57]	1.57 [1.13; 1.91]	1.89 [1.05; 3.87]	1.50 [0.97; 2.34]	0.206
S (1, 0) Correlat	0.83 [0.79; 0.87]	0.82 [0.81; 0.86]	0.87 [0.79; 0.91]	0.84 [0.80; 0.89]	0.85 [0.76; 0.88]	0.797
S (0, 1) Correlat	0.87 [0.82; 0.90]	0.84 [0.82; 0.87]	0.89 [0.87; 0.92]	0.89 [0.86; 0.91]	0.87 [0.84; 0.91]	0.283
S (1, 1) Correlat	0.77 [0.68; 0.81]	0.75 [0.72; 0.79]	0.82 [0.73; 0.87]	0.79 [0.72; 0.84]	0.76 [0.65; 0.85]	0.685
S (1, −1) Correlat	0.76 [0.70; 0.81]	0.75 [0.72; 0.79]	0.83 [0.77; 0.84]	0.77 [0.74; 0.83]	0.80 [0.71; 0.84]	0.692
S (2, 0) Correlat	0.61 [0.49; 0.68]	0.62 [0.58; 0.71]	0.72 [0.56; 0.77]	0.63 [0.50; 0.72]	0.66 [0.51; 0.71]	0.888
S (0, 2) Correlat	0.69 [0.64; 0.77]	0.67 [0.59; 0.70]	0.77 [0.73; 0.79]	0.72 [0.61; 0.77]	0.67 [0.62; 0.80]	0.577
S (2, 2) Correlat	0.43 [0.34; 0.56]	0.50 [0.43; 0.52]	0.60 [0.43; 0.64]	0.44 [0.34; 0.59]	0.47 [0.28; 0.57]	0.921
S (2, −2) Correlat	0.44 [0.33; 0.60]	0.44 [0.42; 0.48]	0.57 [0.44; 0.61]	0.44 [0.35; 0.54]	0.56 [0.38; 0.61]	0.698
S (3, 0) Correlat	0.38 [0.22; 0.53]	0.39 [0.34; 0.51]	0.55 [0.35; 0.59]	0.37 [0.19; 0.51]	0.44 [0.24; 0.50]	0.819
S (0, 3) Correlat	0.51 [0.42; 0.63]	0.38 [0.34; 0.51]	0.62 [0.55; 0.63]	0.50 [0.34; 0.60]	0.47 [0.40; 0.67]	0.565
S (3, 3) Correlat	0.15 [0.03; 0.31]	0.20 [0.17; 0.25]	0.29 [0.13; 0.34]	0.18 [0.02; 0.34]	0.17 [0.05; 0.28]	0.989
S (3, −3) Correlat	0.23 [0.07; 0.37]	0.18 [0.14; 0.21]	0.25 [0.18; 0.30]	0.17 [0.03; 0.28]	0.34 [0.10; 0.46]	0.418
SumOfSqs	1.57 [0.86; 3.28]	1.27 [0.75; 4.91]	1.75 [1.16; 2.73]	2.33 [1.33; 4.38]	2.12 [1.35; 4.37]	0.330
InvDfMom	0.67 [0.59; 0.77]	0.69 [0.62; 0.73]	0.63 [0.60; 0.68]	0.59 [0.49; 0.69]	0.64 [0.59; 0.70]	0.095
SumAverg	51.1 [44.6; 57.6]	51.0 [42.9; 54.2]	45.7 [37.4; 53.6]	50.6 [44.7; 56.5]	56.0 [48.4; 61.1]	0.729
SumVarnc	5.11 [2.74; 10.4]	4.11 [2.27; 15.8]	5.42 [3.15; 9.37]	7.63 [4.18; 12.7]	7.33 [3.96; 15.0]	0.374
SumEntrp	0.88 [0.74; 1.01]	0.85 [0.75; 0.92]	0.92 [0.82; 1.01]	0.97 [0.85; 1.10]	0.94 [0.80; 1.08]	0.111
Entropy	1.19 [0.92; 1.36]	1.05 [0.96; 1.26]	1.24 [1.13; 1.31]	1.34 [1.08; 1.57]	1.27 [1.04; 1.41]	0.085
DifVarnc	0.50 [0.32; 0.96]	0.42 [0.37; 1.50]	0.68 [0.49; 0.81]	0.77 [0.49; 1.47]	0.66 [0.46; 0.98]	0.275

Note: angular second moment (AngScMom), contrast (Contrast), correlation (Correlat), sum of squares (SumOfSqs), inverse difference moment (InvDfMom), entropy (Entropy), sum average (SumAverg), sum variance (SumVarnc), sum entropy (SumEntrp), difference variance (DifVarnc), and difference entropy (DifEntrp).

**Table 4:** Comparison between groups of differentiation grade (Kruskal–Wallis test).

Variable	Good (n = 6)		Differentiation Grade Moderate (n = 95)		Poor (n = 19)		*p*-Value
	Mean (S.D.)	Median [Q1; Q3]	Mean (S.D.)	Median [Q1; Q3]	Mean (S.D.)	Median [Q1; Q3]	
AngScMom	0.12 (0.06)	0.09 [0.08; 0.15]	0.11 (0.11)	0.08 [0.05; 0.14]	0.12 (0.09)	0.09 [0.05; 0.17]	0.453
Contrast	1.21 (0.70)	1.12 [0.61; 1.69]	2.55 (2.82)	1.64 [0.81; 3.47]	2.39 (2.58)	1.35 [0.86; 2.44]	0.538
S (1, 0) Correlat	0.84 (0.10)	0.87 [0.82; 0.92]	0.83 (0.09)	0.84 [0.80; 0.89]	0.82 (0.08)	0.84 [0.80; 0.88]	0.729
S (0, 1) Correlat	0.90 (0.04)	0.89 [0.87; 0.92]	0.87 (0.07)	0.88 [0.84; 0.90]	0.86 (0.06)	0.86 [0.83; 0.90]	0.334
S (1, 1) Correlat	0.80 (0.11)	0.83 [0.78; 0.88]	0.76 (0.12)	0.78 [0.71; 0.83]	0.75 (0.10)	0.75 [0.69; 0.82]	0.407
S (1, −1) Correlat	0.79 (0.11)	0.83 [0.76; 0.86]	0.76 (0.12)	0.76 [0.72; 0.82]	0.76 (0.12)	0.79 [0.72; 0.84]	0.571
S (2, 0) Correlat	0.64 (0.24)	0.72 [0.65; 0.79]	0.58 (0.20)	0.62 [0.49; 0.71]	0.60 (0.19)	0.66 [0.55; 0.71]	0.376
S (0, 2) Correlat	0.77 (0.08)	0.77 [0.74; 0.80]	0.69 (0.13)	0.70 [0.62; 0.77]	0.68 (0.12)	0.67 [0.61; 0.74]	0.170
S (2, 2) Correlat	0.54 (0.26)	0.62 [0.57; 0.66]	0.45 (0.21)	0.44 [0.34; 0.58]	0.43 (0.17)	0.50 [0.32; 0.56]	0.227
S (2, −2) Correlat	0.51 (0.22)	0.57 [0.48; 0.63]	0.44 (0.19)	0.44 [0.34; 0.57]	0.46 (0.21)	0.51 [0.42; 0.60]	0.325
S (3, 0) Correlat	0.42 (0.29)	0.55 [0.41; 0.59]	0.35 (0.24)	0.37 [0.20; 0.52]	0.37 (0.22)	0.41 [0.28; 0.50]	0.414
S (0, 3) Correlat	0.61 (0.14)	0.62 [0.60; 0.64]	0.49 (0.17)	0.50 [0.38; 0.62]	0.48 (0.17)	0.46 [0.38; 0.55]	0.200
S (3, 3) Correlat	0.26 (0.30)	0.30 [0.28; 0.40]	0.18 (0.25)	0.17 [0.02; 0.34]	0.17 (0.16)	0.19 [0.10; 0.28]	0.476
S (3, −3) Correlat	0.27 (0.18)	0.25 [0.20; 0.38]	0.18 (0.21)	0.19 [0.04; 0.29]	0.23 (0.22)	0.20 [0.13; 0.41]	0.435
SumOfSqs	1.90 (1.35)	1.74 [0.89; 2.20]	3.33 (4.58)	1.94 [1.13; 3.88]	2.90 (2.60)	1.92 [0.88; 4.56]	0.800
InvDfMom	0.68 (0.09)	0.67 [0.62; 0.76]	0.62 (0.14)	0.61 [0.53; 0.73]	0.64 (0.14)	0.65 [0.58; 0.73]	0.502
SumAverg	46.0 (14.0)	45.7 [33.7; 56.7]	50.6 (11.6)	50.6 [44.6; 57.2]	50.9 (11.7)	52.5 [42.9; 58.6]	0.719
SumVarnc	6.40 (4.88)	5.42 [2.73; 7.73]	10.8 (15.8)	5.96 [3.60; 12.2]	9.19 (8.30)	6.05 [2.65; 15.4]	0.830
SumEntrp	0.89 (0.15)	0.91 [0.78; 0.99]	0.92 (0.22)	0.94 [0.81; 1.06]	0.90 (0.20)	0.88 [0.76; 1.04]	0.784
Entropy	1.14 (0.22)	1.20 [0.98; 1.26]	1.24 (0.36)	1.27 [1.00; 1.48]	1.20 (0.34)	1.19 [0.96; 1.39]	0.595
DifVarnc	0.51 (0.26)	0.46 [0.29; 0.74]	1.01 (1.02)	0.65 [0.38; 1.28]	1.01 (1.01)	0.53 [0.40; 1.02]	0.522

Note: S.D.: standard deviation; Q1: first quartile; Q3: third quartile. angular second moment (AngScMom), contrast (Contrast), correlation (Correlat), sum of squares (SumOfSqs), inverse difference moment (InvDfMom), entropy (Entropy), sum average (SumAverg), sum variance (SumVarnc), sum entropy (SumEntrp), difference variance (DifVarnc), and difference entropy (DifEntrp).

**Table 5:** Comparison between groups of HPV status (Mann–Whitney test) (own authorship).

Variable	Negative (n = 49)		Positive (n = 71)		*p*-Value
	Mean (S.D.)	Median [Q1; Q3]	Median (S.D.)	Median [Q1; Q3]	
AngScMom	0.15 (0.13)	0.10 [0.06; 0.17]	0.09 (0.07)	0.07 [0.04; 0.12]	0.003
Contrast	1.93 (2.07)	1.03 [0.68; 2.20]	2.83 (3.05)	1.81 [0.99; 3.60]	0.016
S (1, 0) Correlat	0.83 (0.07)	0.83 [0.80; 0.87]	0.83 (0.10)	0.84 [0.80; 0.89]	0.273
S (0, 1) Correlat	0.86 (0.06)	0.86 [0.82; 0.90]	0.87 (0.07)	0.89 [0.85; 0.91]	0.079
S (1, 1) Correlat	0.76 (0.10)	0.77 [0.69; 0.81]	0.76 (0.12)	0.79 [0.71; 0.84]	0.287
S (1, −1) Correlat	0.76 (0.10)	0.75 [0.70; 0.81]	0.76 (0.13)	0.79 [0.74; 0.83]	0.276
S (2, 0) Correlat	0.60 (0.17)	0.62 [0.52; 0.70]	0.58 (0.22)	0.64 [0.50; 0.72]	0.981
S (0, 2) Correlat	0.69 (0.11)	0.69 [0.63; 0.74]	0.69 (0.14)	0.72 [0.62; 0.77]	0.652
S (2, 2) Correlat	0.45 (0.18)	0.45 [0.36; 0.56]	0.45 (0.22)	0.45 [0.32; 0.59]	0.887
S (2, −2) Correlat	0.46 (0.19)	0.44 [0.36; 0.60]	0.44 (0.20)	0.46 [0.35; 0.57]	0.731
S (3, 0) Correlat	0.37 (0.22)	0.39 [0.26; 0.53]	0.34 (0.25)	0.38 [0.20; 0.51]	0.522
S (0, 3) Correlat	0.50 (0.15)	0.51 [0.39; 0.60]	0.49 (0.17)	0.50 [0.38; 0.63]	0.875
S (3, 3) Correlat	0.18 (0.23)	0.18 [0.04; 0.29]	0.19 (0.24)	0.18 [0.02; 0.33]	0.985
S (3, −3) Correlat	0.21 (0.22)	0.21 [0.10; 0.35]	0.18 (0.21)	0.20 [0.03; 0.29]	0.401
SumOfSqs	2.55 (2.93)	1.53 [0.79; 3.16]	3.63 (4.89)	2.32 [1.29; 4.27]	0.034
InvDfMom	0.67 (0.14)	0.69 [0.59; 0.76]	0.60 (0.13)	0.60 [0.49; 0.69]	0.006
SumAverg	49.8 (12.7)	51.0 [43.9; 57.5]	50.8 (11.0)	51.2 [43.9; 56.9]	0.763
SumVarnc	8.28 (10.0)	5.08 [2.37; 10.2]	11.7 (16.9)	7.49 [4.15; 13.6]	0.044
SumEntrp	0.85 (0.23)	0.87 [0.73; 0.98]	0.96 (0.19)	0.97 [0.83; 1.09]	0.008
Entropy	1.12 (0.36)	1.13 [0.93; 1.36]	1.31 (0.32)	1.29 [1.08; 1.52]	0.005
DifVarnc	0.80 (0.77)	0.46 [0.33; 0.94]	1.11 (1.12)	0.77 [0.48; 1.33]	0.024

angular second moment (AngScMom), contrast (Contrast), correlation (Correlat), sum of squares (SumOfSqs), inverse difference moment (InvDfMom), entropy (Entropy), sum average (SumAverg), sum variance (SumVarnc), sum entropy (SumEntrp), difference variance (DifVarnc), and difference entropy (DifEntrp). Bold indicates significant *p* value.

## Data Availability

The datasets generated and/or analyzed during the current study are available from the corresponding author upon reasonable request.
